# Targeting Notch signaling as a novel therapy for retinoblastoma

**DOI:** 10.18632/oncotarget.12142

**Published:** 2016-09-20

**Authors:** Laura Asnaghi, Arushi Tripathy, Qian Yang, Harpreet Kaur, Allison Hanaford, Wayne Yu, Charles G. Eberhart

**Affiliations:** ^1^ Department of Pathology, Johns Hopkins University, School of Medicine, Baltimore, MD, USA; ^2^ Department of Ophthalmology, Johns Hopkins University, School of Medicine, Baltimore, MD, USA; ^3^ Department of Oncology, Johns Hopkins University, School of Medicine, Baltimore, MD, USA; ^4^ Microarray Core Facility, Sidney Kimmel Cancer Center, Johns Hopkins University, School of Medicine, Baltimore, MD, USA

**Keywords:** Notch, retinoblastoma, proliferation, melphalan, γ-secretase inhibitors

## Abstract

Retinoblastoma is the most common intraocular malignancy of childhood. Notch plays a key role in retinal cells from which retinoblastomas arise, and we therefore studied the role of Notch signaling in promoting retinoblastoma proliferation. Moderate or strong nuclear expression of Hes1 was found in 10 of 11 human retinoblastoma samples analyzed immunohistochemically, supporting a role for Notch in retinoblastoma growth. Notch pathway components were present in WERI Rb1 and Y79 retinoblastoma lines, with Jag2 and DLL4 more highly expressed than other ligands, and Notch1 and Notch2 more abundant than Notch3. The cleaved/active form of Notch1 was detectable in both lines. Inhibition of the pathway, achieved using a γ-secretase inhibitor (GSI) or by downregulating Jag2, DLL4 or CBF1 using short hairpin RNA, potently reduced growth, proliferation and clonogenicity in both lines. Upregulation of *CXCR4* and *CXCR7* and downregulation of *PI3KC2β* were identified by microarray upon Jag2 suppression. The functional importance of *PI3KC2β* was confirmed using shRNA. Synergy was found by combining GSI with Melphalan at their IC_50_. These findings indicate that Notch pathway is active in WERI Rb1 and Y79, and in most human retinoblastoma samples, and suggest that Notch antagonists may represent a new approach to more effectively treat retinoblastoma.

## INTRODUCTION

Retinoblastoma is a malignant tumor of the developing retina, and the most common intraocular cancer in children, with 80% of cases occurring before the age of 3 [1]. It is diagnosed in approximately 300 children per year in the US, with an incidence of 11.8 cases per million in children aged 0–4 years [2]. The age-adjusted incidence rate has remained stable over the last 30 years [2]. Approximately 4,000 deaths related to this malignancy occur annually worldwide [3]. The most common presenting signs are leukocoria, strabismus, and poor vision [4]. Elevated mitotic rate, dystrophic calcification and multiple regions of necrosis are considered microscopic hallmarks [5, 6].

Retinoblastoma is thought to arise from retinal progenitor cells, although a subset may derive from fully differentiated neurons [7]. It has been shown that retinoblastoma cells express markers of postmitotic cone precursors, but not markers of other retinal cell types, supporting a cone precursor origin of retinoblastoma [8]. In addition, a recent study suggested that the diversity of retinoblastoma is a result of tumor progression rather than distinct tumor subtypes, identifying a continuity of tumor phenotypes with a common photoreceptor origin [9]. Considering the role of Notch1 receptor in suppressing photoreceptor differentiation and maintaining their progenitor state [10], Notch pathway activity might be important in promoting survival and inhibiting differentiation of the retinoblastoma-originating cells.

In most children with retinoblastoma, the disease affects only one eye, but in one third tumors grow in both eyes. Bilateral retinoblastomas are generally heritable, while unilateral tumors are not [4]. Cure rates are high if treatment starts early. Current treatment options focus on systemic and/or local chemotherapy and enucleation, but cryotherapy, laser photocoagulation and various types of radiotherapy including external beam radiation therapy (EBR), proton beam therapy and brachytherapy have also been used [4]. Aggressive retinoblastoma can invade the optic nerve and disseminate through the subaracnoid space to the brain and the spinal cord, or invade the choroid and spread hematogenously. These high risk features predicting a worse prognosis often prompt more aggressive systemic treatment [11].

Genetically, the cancer is initiated by the biallelic loss of the oncosuppressor gene *RB1*, which encodes a protein, pRb, known to inhibit cell cycle progression by interacting, in the hypophosphorylated active form, with critical regulatory proteins, including the E2F family of transcription factors [12–14]. Recently, several studies have found additional tumor-suppressor functions for the multifunctional protein pRb, including maintenance of genomic stability and regulation of epigenetic processes, such as DNA methylation, histone modification, and ATP-dependent chromatin reorganization [14–16]. However, the complete mechanisms underlying retinoblastoma initiation and progression still need further elucidation.

Here, we investigated the role of Notch signaling in retinoblastoma, since Notch receptors play an important role during retinal development by inhibiting photoreceptor differentiation and maintaining their progenitor state [10]. Notch signaling is induced by interaction of Jagged (Jag1-2) and Delta-like (DLL1,3,4) ligands with a Notch receptor on an adjacent cell, promoting several successive proteolytic cleavages of the receptor, culminating in the release of the Notch intracellular domain (NICD) mediated by the **γ**-secretase complex. NICD translocates in the nucleus, where it forms a complex with CBF1 and MAML [17, 18], which activates the transcription of Notch target genes, including *Hairy and enhancer of split (Hes)* and *Hes-related repressor protein (Hey)* gene families [19]. These proteins negatively regulate the transcription of genes involved in cellular differentiation. Abnormal Notch pathway activity has been linked to tumorigenesis in many different tumor types, but thus far it has not been extensively studied in retinoblastoma.

During early development of the mammalian retina, Notch1 and Notch3 are expressed in the central portion, while Notch2 is mostly present in the periphery and in the retinal pigmented epithelium [20, 21]. Importantly, gene expression profile analysis performed in human retinoblastoma samples [22] and in a murine retinoblastoma model [23] indicated that Notch pathway components are differentially expressed in retinoblastoma tissues as compared to normal retina. Here we demonstrate protein expression of the Notch target Hes1 in primary tumors samples, and a functional role for Notch signaling in retinoblastoma cell lines, suggesting that Notch pathway represents a potential new target for the treatment of these aggressive childhood tumors.

## RESULTS

### Expression of Notch pathway components in retinoblastoma lines and primary tumors

**Figure 1:**
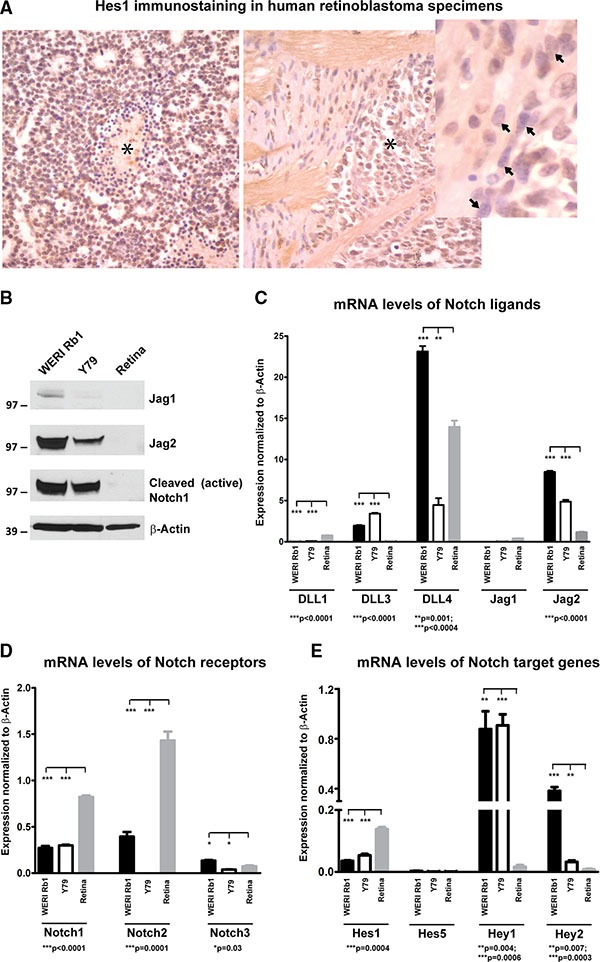
Expression of Notch pathway components in retinoblastoma primary tumors and cell lines (**A**) Nuclear Hes1 immunoreactivity was examined in 11 human retinoblastoma tumors by immunohistochemistry. The left panel shows strong diffuse immunoreactivity in tumor cells, with some negative dying cells present around a central region of necrosis (asterisk). The right side panel shows Hes1 positive tumor cells (asterisk) invading the optic nerve head. In the inset, spindled stromal cells in this region are negative for Hes1 (arrow). (**B**) Elevated protein levels of Jag2 and cleaved Notch1 receptor were found by Western blot in WERI Rb1 and Y79 lines, as compared to non-neoplastic adult retina. However, minimal Jag1 protein expression was observed in both retinoblastoma lines and in non-neoplastic adult retina; β-Actin was used as a loading control. (**C**) mRNA levels of the Notch ligands Jag2 and DLL4 were much higher as compared to Jag1 and DLL1,3 in WERI Rb1 and Y79 lines, as found by qPCR. Normal adult retina although expressed high levels of DLL4 mRNA as compared to the other ligands. (**D**) Notch1 and 2 mRNA levels were more elevated than Notch3 in WERI Rb1 cells, whereas Notch1 was more expressed than Notch2 and 3 in Y79 cells. Notch1 and 2 mRNA levels were also highly expressed in the non-neoplastic adult retina, as determined by qPCR. (**E**) mRNA levels of the Notch target genes were determined by qPCR in WERI Rb1, Y79, and in non-neoplastic adult retina. Hey1 was the most highly expressed Notch target gene in both retinoblastoma lines, while Hes1 was the most represented Notch target gene in the non-neoplastic adult retina.

Expression of the Notch target Hes1 was evaluated by immunohistochemistry in 11 human retinoblastoma samples (Figure 1A). Nuclear Hes1 immunoreactivity was present in all tumors examined, with strong expression in 7 (64%), moderate expression in 3 (27%), and weak staining in 1 (9%). Non-neoplastic stromal elements served as internal negative controls. We next analyzed expression levels of Notch pathway components in WERI Rb1 and Y79 retinoblastoma cell lines. We found by Western blot that Jag2 ligand was highly expressed in both lines, but not detectable in protein extracts from normal adult retina (Figure 1B). Interestingly, Jag2 mRNA and protein levels were much higher in both retinoblastoma lines as compared to Jag1, which was barely measurable by both Western blot and qPCR (Figure 1B, 1C). The cleaved and active form of Notch1 receptor (NICD1) was also highly expressed in both retinoblastoma lines, but not detectable in the normal adult retina, indicating that Notch pathway is active in these retinoblastoma lines under standard culture conditions (Figure 1B).

Among the DLL ligands, DLL4 mRNA appeared to be highly expressed in both retinoblastoma lines, compared to DLL1 and DLL3, and it was also highly expressed in the non-neoplastic adult retina (Figure 1C). Among the receptors, Notch1 and 2 appeared to be more abundant at the transcriptional level than Notch3 in WERI Rb1, while Notch1 was more highly expressed than Notch2 and 3 in Y79. However, Notch1 and 2 were also highly expressed in the normal adult retina (Figure 1D). Regarding Notch target genes, Hey1 mRNA levels were 2 to 8 fold higher as compared to other target genes in both lines, while Hes1 was the most highly expressed Notch target in the normal adult retina (Figure 1E). These data indicate that Notch pathway is active in WERI Rb1 and Y79 lines as well as in most of the human retinoblastoma samples that we analyzed.

### Pharmacological blockade of Notch pathway inhibits cell growth and clonogenicity in retinoblastoma lines

**Figure 2:**
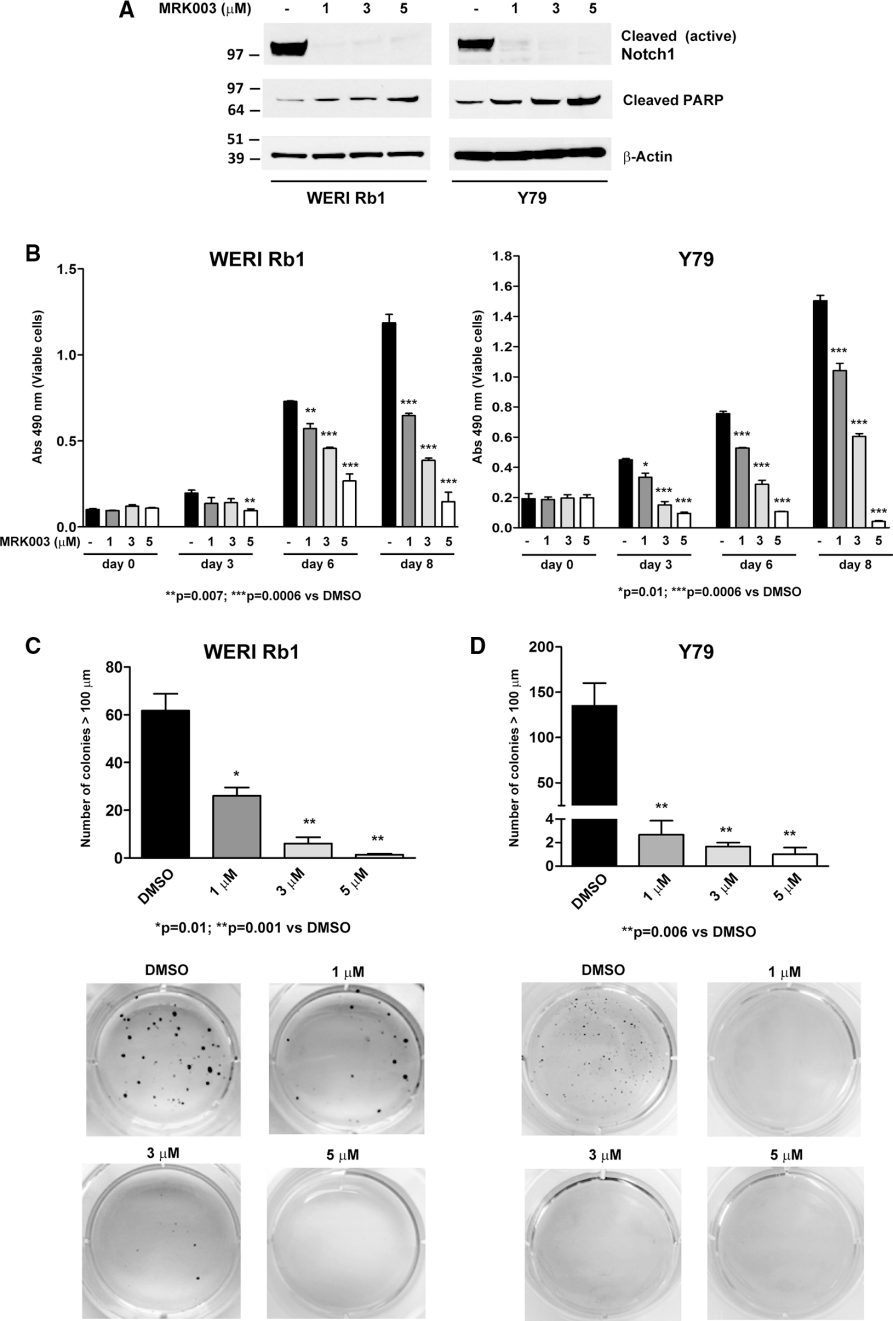
Pharmacological Notch blockade inhibits growth and clonogenicity in retinoblastoma lines (**A**) Inhibition of the cleavage of Notch1 receptor was determined by Western blot in WERI Rb1 and Y79 cells treated with MRK003 at 1, 3, and 5 μM for 72 hours as compared to DMSO vehicle control, using an antibody that recognizes the cleaved/active form of the Notch1 intracellular domain (NICD1). A dose-dependent increase of the apoptotic marker cleaved PARP was observed through Western blot by treating both lines with MRK003 at 1, 3, and 5 μM for 72 hours, using β-Actin as a loading control. (**B**) Cell growth was determined by CCK-8 growth assay after 0, 3, 6, and 8 days of treatment with MRK003 at 1, 3, and 5 μM in both lines. (**C**), (**D**) Clonogenic growth in soft agar was significantly reduced in a dose-dependent fashion after treating WERI Rb1 (C) and Y79 (D) cells with the Notch pathway inhibitor MRK003 at 1, 3, and 5 μM for 30 days, as determined by soft agar assay. Experiments were performed in triplicates. Data are presented as mean ± SD.

Given the evidence of active Notch signaling under standard culture conditions in WERI Rb1 and Y79 lines, we blocked the pathway using the γ-secretase inhibitor (GSI) MRK003 [24] and assessed the effects on tumor growth. Inhibition of activating cleavage of the Notch 1 receptor was noted after MRK003 was added at 1, 3, and 5 μM, as determined by the reduction of NICD1 levels after 72 hours (Figure 2A). In parallel to Notch signaling inhibition, we found a dose-dependent increase in the cleavage of PARP, a marker of apoptosis, when treating both lines with MRK003 (Figure 2A), suggesting that pharmacological blockade of Notch induced apoptosis in these lines. In addition to activation of apoptosis, we observed a dose-dependent inhibition in growth upon treatment with MRK003 for 3, 6, and 8 days, with reductions of 50 to 70% in WERI Rb1 and 30 to 80% in Y79 at day 8 (Figure 2B).

Interestingly, we observed an even more remarkable reduction in clonogenic growth in soft agar after pharmacologically suppressing the Notch pathway in both retinoblastoma lines (Figure 2C, 2D). WERI Rb1 cells treated with DMSO vehicle control formed an average of 61.7 ± 7.1 colonies with diameter greater than 100 μm, whereas WERI Rb1 cells treated with MRK003 at 1, 3 or 5 μM generated respectively an average of 26.0 ± 3.5, 6.0 ± 2.6, and 1.3 ± 0.3 colonies after 30 days of incubation in soft agar, thus increasing levels of the GSI reduced the number of clonogenic capacity by 58%, 90% and 98% respectively as compared to DMSO. Similarly, Y79 cells treated with DMSO formed an average of 135.0 ± 25.0 colonies with diameter greater than 100 μm, whereas Y79 cells treated with MRK003 at 1, 3 or 5 μM produced respectively an average of 2.7 ± 1.2, 1.7 ± 0.3, and 1.0 ± 0.6 colonies corresponding to 98.0%, 98.7% and 99.2% reductions. These values were statistically significant in both cell lines with all doses of MRK003, as shown in Figure 2C, 2D. However, we did not find any significant inhibition of invasion through Matrigel by treating both lines with MRK003 at 1, 3 or 5 μM (Supplementary Figure S1). These data indicate that pharmacological blockade of Notch signaling potently reduced cellular and clonogenic growth of retinoblastoma cells. However, treatment with MRK003 at 1, 3 or 5 μM did not significantly decrease growth in a non-transformed retinal precursor cell line (R28), as found by MTS growth assay (Supplementary Figure S2).

### Genetic downregulation of the Notch pathway inhibits growth and proliferation of retinoblastoma lines

**Figure 3:**
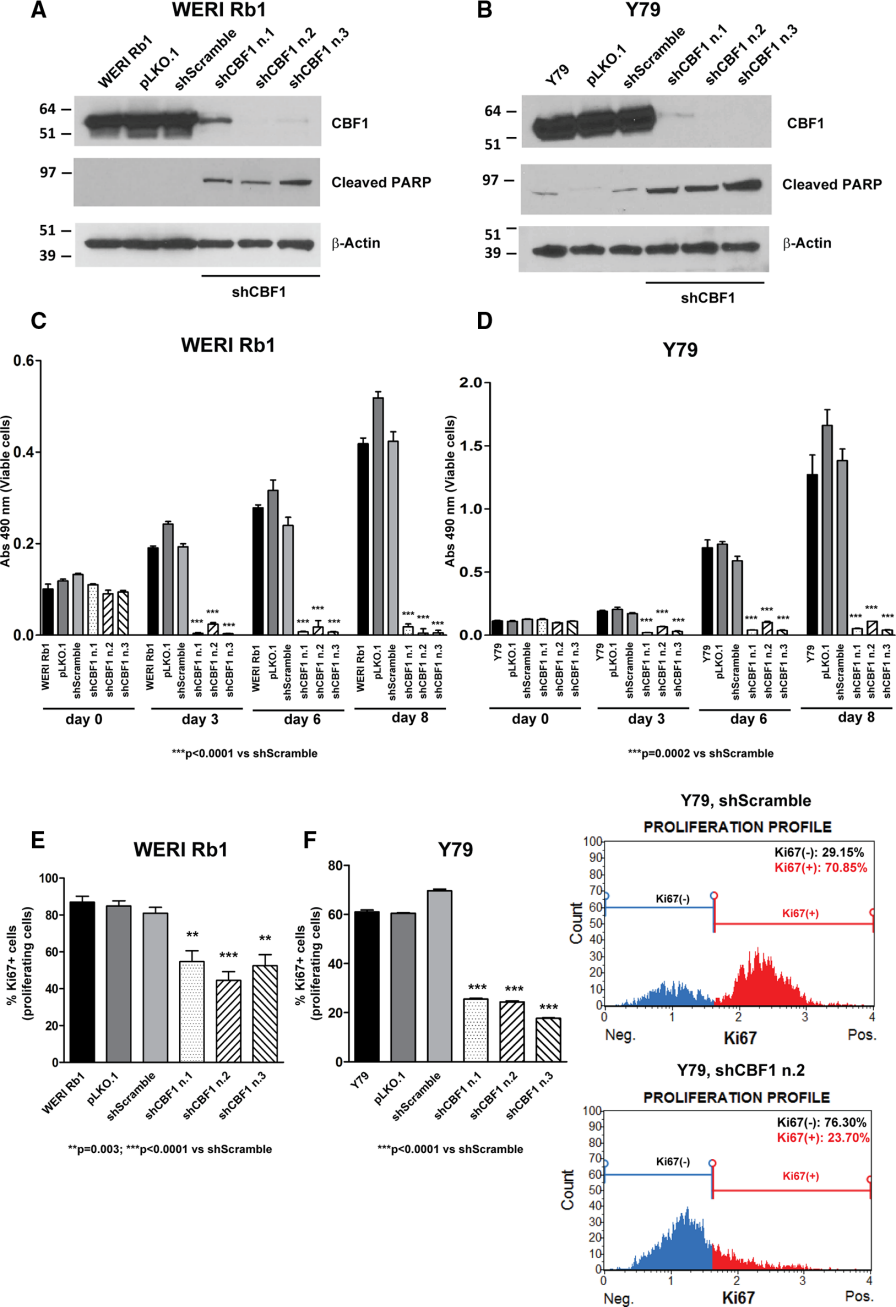
Genetic downregulation of CBF1 inhibits growth and proliferation of retinoblastoma cells (**A**), (**B**) The protein expression of CBF1, a downstream effector of the Notch pathway, was suppressed by three separate shRNA constructs in WERI Rb1 (A) and Y79 (B) cells, compared to pLKO.1 vector or scrambled shRNA or parental cells, as found by Western blot. Increase in cleaved PARP was also observed by Western blot in WERI Rb1 and Y79 cells upon CBF1 suppression, using β-Actin as a loading control. (**C**), (**D**) Downregulation of CBF1 reduced cell growth by 85 to 90% with all three CBF1 shRNA constructs in WERI Rb1 (C) and Y79 (D) cells, compared to scrambled shRNA control, as determined by CCK-8 growth assay. (**E**), (**F**) All three CBF1 shRNA constructs reduced proliferation by 30 to 45% in WERI Rb1 (E) and by 60 to 75% in Y79 (F) cells, compared to scrambled shRNA infected cells, as found by Ki67 incorporation assay. Experiments were performed in triplicates. Data are presented as mean ± SD. The graphs in the right part of panel F are representative images showing reduction in Ki67^+^ cells upon suppression of CBF1, compared to scrambled shRNA control.

To further investigate the role of Notch in promoting retinoblastoma growth, we genetically inhibited the pathway using shRNAs that specifically target CBF1, a critical cofactor in canonical Notch signaling that binds NICD in the nucleus and mediates Notch target gene transcription. Three separate short hairpin RNA (shRNA) constructs reduced protein expression of CBF1 by more than 80% on Western blot, in both WERI Rb1 (Figure 3A) and Y79 cells (Figure 3B). Induction of the apoptotic marker cleaved PARP was observed in both lines with all three CBF1 shRNA constructs, as compared to pLKO.1 or scrambled shRNA infected cells or the parental lines (Figure 3A, 3B). A profound reduction in overall cell growth by 85 to 90% was noted with all three CBF1 shRNA constructs in both cell lines, as compared to pLKO.1 vector or scrambled shRNA (Figure 3C, 3D). Proliferation was also reduced by inhibiting CBF1 expression. The percentage of cells positive for Ki67 was reduced by 30 to 45% in WERI Rb1 (Figure 3E) and by 60 to 75% in Y79 cells with all three CBF1 shRNA constructs as compared to scrambled shRNA (Figure 3F).

**Figure 4:**
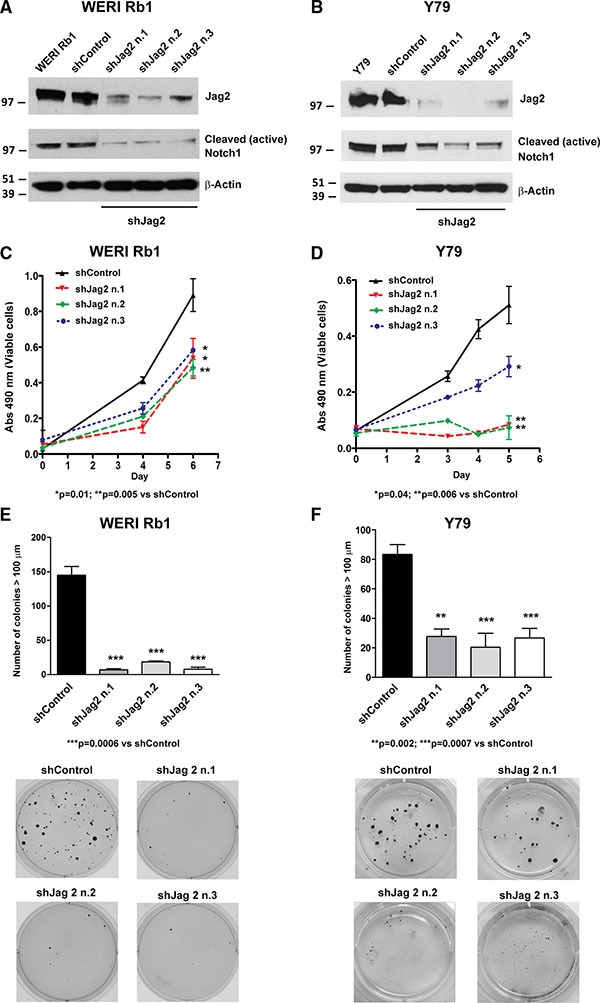
Genetic downregulation of the Notch ligand Jag2 inhibits growth and proliferation of retinoblastoma cells (**A**), (**B**) The protein expression of Jag2 ligand was suppressed by three separate shRNA constructs in WERI Rb1 (A) and Y79 (B) cells, compared to shRNA control or parental cells, as found by Western blot. The protein levels of the cleaved/active form of the Notch1 intracellular domain (NICD1) were also reduced in the cells expressing Jag2 shRNAs as compared to shRNA control or parental cells, indicating inhibition of Notch signaling by Jag2 downregulation. (**C**), (**D**) Cell growth was significantly reduced in WERI Rb1 (C) and Y79 (D) cells expressing Jag2 shRNAs compared to shRNA controls, as found by CCK-8 growth assay. (**E**), (**F**) Inhibition of clonogenic growth was observed in WERI Rb1 (E) and Y79 (F) cells expressing Jag2 shRNAs compared to shRNA controls, as determined by soft agar assay. Experiments were performed in triplicates. Data are presented as mean ± SD. The microphotographs in the lower part of panels E and F are representative images of the soft agar colonies after 30 days in culture.

Since we observed that Jag2 mRNA and protein levels were more elevated than Jag1 in both retinoblastoma lines, and also upregulated compared to normal adult retina (Figure 1B, 1C), we decided to also genetically inhibit Notch signaling by downregulating expression of this Notch ligand. Three shRNA constructs targeting Jag2 produced approximately 80% reduction in protein expression in WERI Rb1 cells (Figure 4A) and 90% reduction in Y79 cells (Figure 4B). A profound concomitant decrease in NICD1 protein levels indicated that receptor activation had been inhibited by Jag2 downregulation (Figure 4A, 4B). Next we analyzed the effects that blocking this key upstream driver of signaling might have on growth, clonogenicity and invasion. We found that the suppression of Jag2 ligand significantly reduced cell growth by about 50% in WERI Rb1 and from 60 to 70% in Y79, as determined by CCK-8 assay (Figure 4C, 4D). However, we observed an even more potent inhibitory effect on clonogenic growth, as all three shJag2 constructs suppressed colony formation in soft agar from 87 to 95% in WERI Rb1 and from 67 to 76% in Y79 cells, compared to control shRNA (Figure 4E, 4F). These data support a role for Jag2 in promoting cell growth and clonogenicity in retinoblastoma cells. We did not observe any inhibition of invasion by suppressing Jag2 (Supplementary Figure S3).

Since DLL4 ligand was highly expressed in both retinoblastoma cell lines as well as in the non-neoplasitc adult retina, we genetically inhibited its expression in the retinoblastoma lines by shRNAs. Two shRNA contructs, TRCN0000033415 (n.1) and TRCN0000033418 (n.2), reduced by more than 70% the mRNA levels of DLL4 in both retinoblastoma lines, as compared to scrambled shRNA (Supplementary Figure S4A). This reduction was associated with a significant inhibition in growth, which was reduced from 60 to 80% in WERI Rb1 (Supplementary Figure S4B) and by about 50% in Y79, as found by CCK-8 assay (Supplementary Figure S4C), further supporting a role for ligand-driven Notch signaling in promoting retinoblastoma growth.

### Notch inhibition increases the apoptotic effect of melphalan in retinoblastoma cells

**Figure 5:**
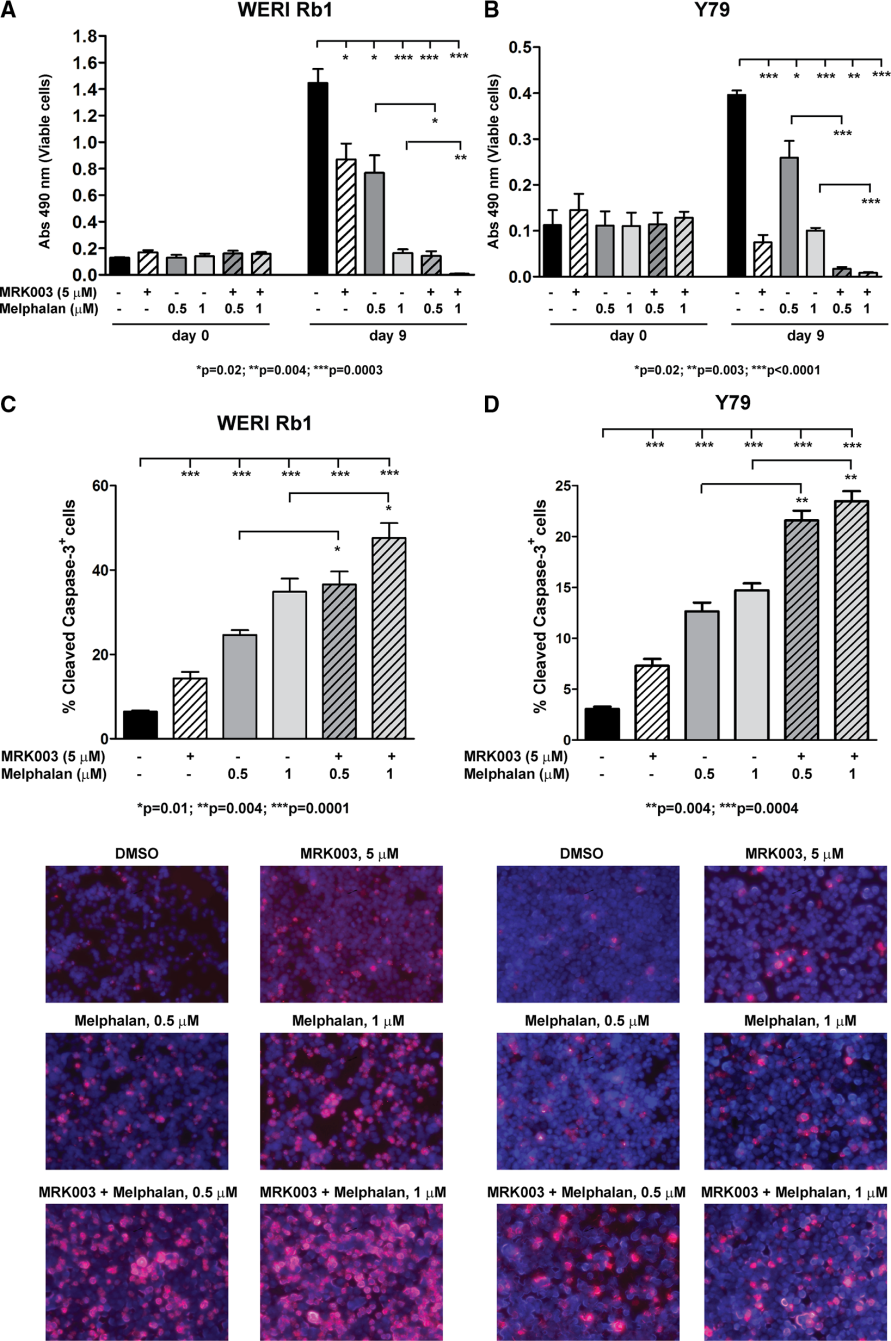
MRK003 enhances the growth inhibitory and pro-apoptotic effects of Melphalan in retinoblastoma cells (**A**) Combinatorial treatment of MRK003 (5 μM) and Melphalan (0.5 and 1 μM) for 9 days was more effective in suppressing growth as compared to Melphalan alone, both in WERI Rb1 (A) and in Y79 (**B**) cells, as determined by CCK-8 growth assay. Experiments were performed in triplicates. Data are presented as mean ± SD. (**C**, **D)** Combinatorial treatment of MRK003 (5 μM) and Melphalan (0.5 and 1 μM) for 5 days significantly increased apoptosis as compared to monotherapy, both in WERI Rb1 (C) and in Y79 (D) cells, as determined by immunofluorescence assay using an antibody specific for cleaved caspase-3 (red), a marker of apoptosis. Nuclei were stained with DAPI (blue). Microphotographs in the lower part of panels C and D are representative images of the immunofluorescence staining.

One of the most effective treatment options for retinoblastoma is systemic or local chemotherapy. Since Melphalan is often used as a therapy in children with retinoblastoma, we decided to investigate whether the combination of Melphalan with the GSI MRK003 was more effective in reducing cell viability and inducing apoptotic cell death than single agents. WERI Rb1 and Y79 cells were treated with Melphalan at 0.5 or 1 μM alone or in combination with 5 μM of MRK003. DMSO-treated cells were used as control. CCK-8 growth assays showed that combinatorial treatment of 5 μM of MRK003 with Melphalan at 0.5 or 1 μM significantly reduced by more than 70% growth in both lines after 9 days of culture as compared to Melphalan alone (Figure 5A, 5B). We also measured activation of the apoptotic pathway after 5 days of treatment by determining, through immunofluorescence, the percentage of cells positive for cleaved caspase-3, a marker of late apoptosis. Interestingly we observed a significant increase in the percentage of cells positive for cleaved caspase-3 when the chemotherapeutic agent was administered in association with the Notch pathway inhibitor MRK003, as compared to single agent treatment (Figure 5C, 5D). In particular, the association of Melphalan 0.5 or 1 μM with MRK003 at 5 μM produced an increase of 48% and 36% respectively in the percentage of WERI Rb1 cells positive for cleaved caspase-3 (*p* = 0.01, Figure 5C), and an increase of 70% and 60% respectively in Y79 cells positive for cleaved caspase-3 (*p* = 0.004), compared to treatment with Melphalan alone (Figure 5D).

**Figure 6:**
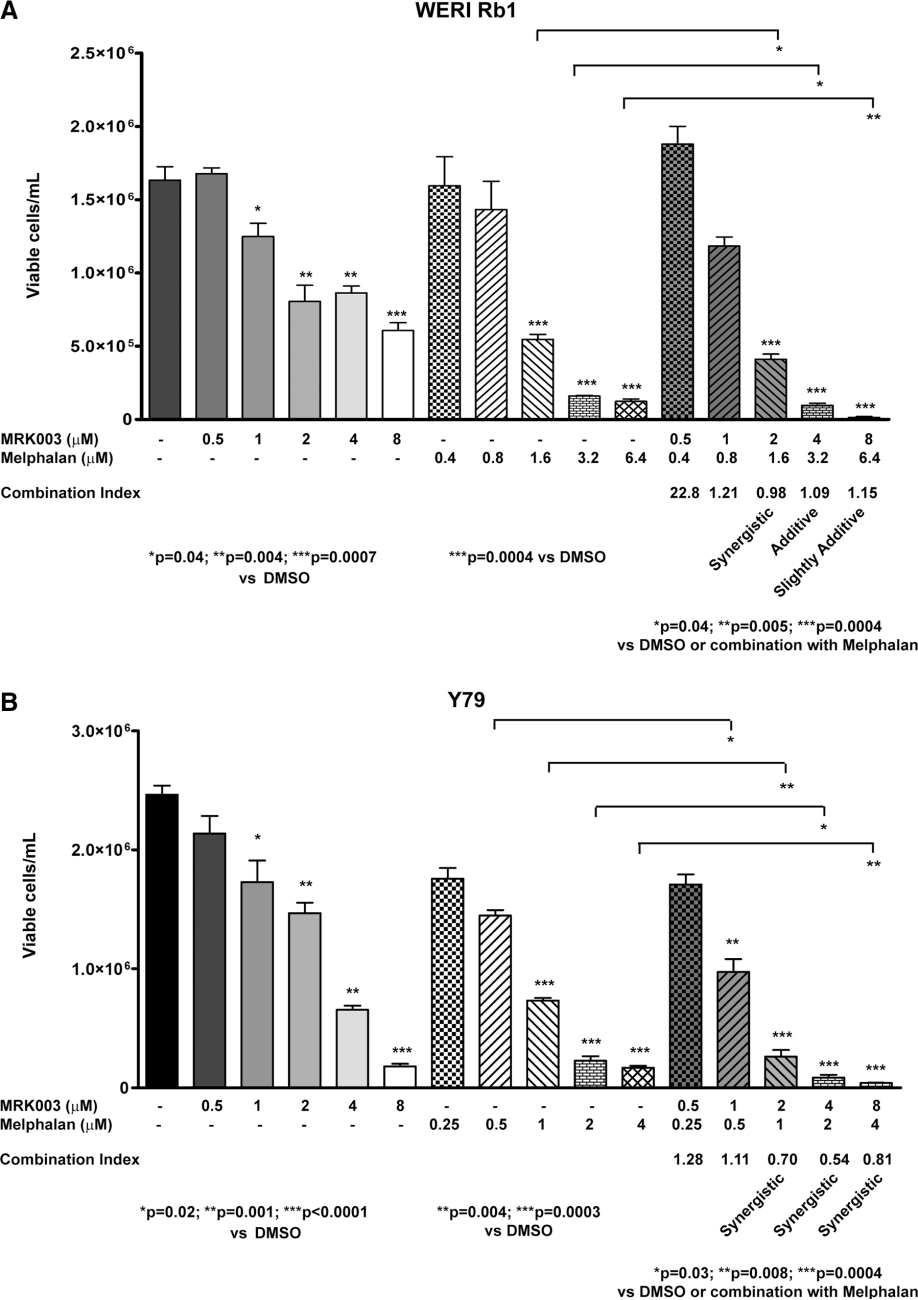
Combination of Melphalan with Notch pathway inhibition had at least an additive effect in reducing cell viability in retinoblastoma (**A**), (**B**) WERI Rb1 (A) and Y79 (B) cells were treated for 5 days with serial dilutions of the two drugs alone or in combination, and the number of viable cells was determined by Trypan Blue Exclusion Dye. Each condition was performed in biological triplicates. Data are presented as mean ± SD. The fraction of viable cells was used to calculate the Combination Index (CI) using CompuSyn software: CI < 1, =1, >1 indicate respectively synergistic, additive, and antagonistic effects [53].

In order to define the interaction between the two drugs more in detail, we used CompuSyn software. We performed serial dilutions of the chemotherapeutic agent alone or in combination with MRK003, counting the number of viable cells/mL with trypan blue exclusion dye, after 5 days of treatment. We found that the combination of Melphalan with MRK003 had at least an additive effect in reducing the number of viable WERI Rb1 cells, after 5 days of culture, compared to single agent therapy, at concentrations of 1.6, 3.2, 6.4 μM of Melphalan and respectively 2, 4, 8 μM of MRK003, with a combination index (CI) < 1 (Figure 6A). In Y79 we found that such combinations had a synergistic effect in reducing the number of viable cells, after 5 days of culture at concentrations of 1, 2, 4 μM of Melphalan and 2, 4, 8 μM of MRK003, with CI < 1 by combining these concentrations (Figure 6B). Interestingly the combinatorial treatment produced a synergistic effect in both lines when the two drugs were used at concentrations equal to their IC_50_, since we obtained CI < 1 when combining MRK003 at 2 μM with Melphalan at 1.6 μM in WERI Rb1 or 1 μM in Y79 (Figure 6A, 6B). We used previously published IC_50_ values of Melphalan for these lines [25].

### Notch blockade enhances the inhibitory effect of γ-radiation on cell survival in retinoblastoma cells

**Figure 7:**
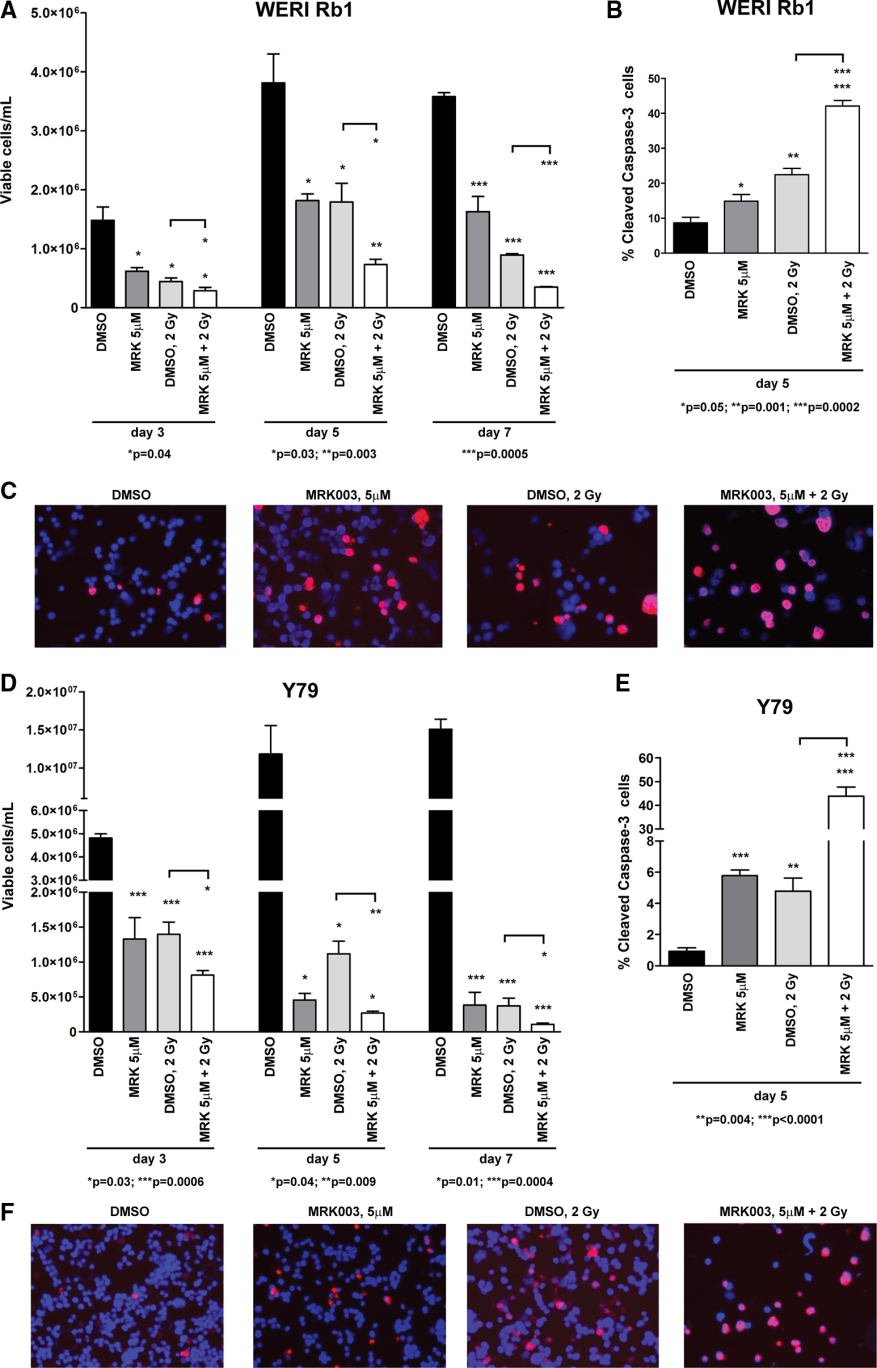
Notch blockade enhances the γ-radiation-dependent inhibition of cell survival in retinoblastoma cells (**A**), (**D**) WERI Rb1 (A) and Y79 (D) cells were treated with 5 μM of MRK003 or DMSO for 2 hours and then exposed to 0 or 2 Gy of γ-radiation in a single dose. Viable cells were counted 3, 5, 7 days after exposure to γ-radiation, by trypan blue exclusion dye. When radiotherapy was combined with MRK003, cell viability was significantly reduced compared to γ-radiation alone, as determined by 2-sided Student *t* test. Experiments were performed in triplicate. Data are presented as mean ± SD. (**B**), (**C**), (**E**), (**F**) Combinatorial treatment of MRK003 (5 μM) and γ-radiation (2 Gy) for 5 days significantly increased apoptosis as compared to single agent therapy, both in WERI Rb1 (B, C) and in Y79 (E, F) cells, as determined by immunofluorescence assay using an antibody specific for cleaved caspase-3 (red), a marker of apoptosis. Nuclei were stained with DAPI (blue). Microphotographs in C and F are representative images of the immunofluorescence staining.

Radiotherapy, including external beam radiation therapy (EBR), proton beam therapy and brachytherapy, has also been used to treat children with retinoblastoma, although generally only after failure of other treatments. Although EBR is an effective treatment modality, with high cure rates [26], it has significant side effects, with children younger than 1 year being particularly susceptible to cataract and increased mortality from radiation-induced secondary neoplasms [27]. Finding new strategies to reduce doses of radiation may help to minimize these adverse reactions. Increasing evidence suggests that Notch signaling plays an important role in mediating radiation resistance in tumor cells [28]. Therefore, we combined exposure to γ-radiation with the GSI MRK003 in retinoblastoma cells to investigate whether Notch blockade might increase the apoptotic effect of radiation. WERI Rb1 and Y79 cells are known to be radiosensitive [29]. We treated both lines with MRK003 at 5 μM or DMSO and exposed them to 0 or 2 Gy of γ-radiation in a single dose. Cell viability was assessed 3, 5, and 7 days post-irradiation, by trypan blue exclusion dye. Apoptosis was determined by cleaved caspase-3 immunofluorescence assay 5 days after irradiation. The combined treatment was more effective in reducing viable cell concentration and increasing apoptosis than either agent alone in both WERI Rb1 (Figure 7A–7C) and Y79 (Figure 7D–7F) lines, reinforcing the role of Notch signaling in promoting retinoblastoma cell survival and growth, and indicating potentiality for a combined regimen.

### Gene expression profile of retinoblastoma cells after downregulation of Jag2 ligand

To determine which downstream signaling pathways might be involved in the suppression of growth and clonogenicity upon downregulation of Jag2 ligand in the retinoblastoma cells, microarray analysis was performed on mRNA extracted from WERI Rb1 and Y79 cells transduced with two different Jag2 shRNAs (#1, #2) as compared to shRNA controls and parental lines. We used Ingenuity^®^ Pathway Analysis (IPA^®^) to determine the top signaling pathways modulated by the genes that we found to be overexpressed or downregulated in the retinoblastoma cells where Jag2 was inhibited. Combining the data obtained by suppressing Jag2 in both retinoblastoma lines, we observed that the most upregulated pathway was the chemokine signaling (*p* = 1.80 × 10^−2^), with *CXCR4* and *CXCR7* (=*ACKR3*) chemokine receptors as the most upregulated genes in both lines upon suppression of Jag2, as summarized in Table 1. The most downregulated pathway was the Epithelial-to-Mesenchymal Transition (EMT) signaling (*p* = 1.76 × 10^−4^), with *PI3KC2β* and *Jag2* as the most downregulated genes in this pathway (Table 1). Notch signaling overall was also one of the most downregulated pathways.

**Table 1:** Top canonical pathways modulated by the genes overexpressed or downregulated in WERI Rb1 and Y79 cells upon downregulation of Jag2 by shRNA, as found using Ingenuity Pathway Analysis (IPA®). P values were calculated with Fisher's Exact Test. Actual Fold Change (FC) was used to determine up or downregulation.

Top upregulated pathways	
Name	p-value
Chemokine Signaling	1.80E-02
Induction of Apoptosis by HIV 1	2.02E-02
Atherosclerosis Signaling	2.13E-02
Ephrin B Signaling	2.24E-02
Granulocyte Adhesion and Diapedesis	3.18E-02

Top downregulated pathways	
Name	p-value
Regulation of the Epithelial-Mesenchymal Transition Pathway	1.76E-04
Role of p14/p19ARF in Tumor Suppression	4.25E-03
IL-9 Signaling	4.62E-03
Notch Signaling	5.17E-03
MSP-RON Signaling Pathway	5.36E-03

Top upregulated genes (Fold Change)	
Molecules	Fold change
**CXCR4**	**2.102**
**CXCR7**	**2.023**

Top downregulated genes (Fold Change)	
Molecules	Fold change
**PIK3C2β**	**−2.089**
**JAG2**	**−1.809**

Since *PI3KC2β* is known to play a role in neuroblastoma tumorigenesis [30], its suppression might contribute to the potent inhibitory effects that we observed on growth and clonogenicity upon suppressing Jag2 in both retinoblastoma lines. To test this hypothesis in retinoblastoma cells, we first confirmed by qPCR the reduction of PI3KC2β mRNA levels upon suppression of Jag2 ligand in both retinoblastoma lines (Supplementary Figure S5A). Then we downregulated the mRNA levels of PI3KC2β using shRNA. Three different shRNA vectors [TRCN0000002119 (n.1), TRCN0000002120 (n.2), TRCN0000002121 (n.3)] suppressed the expression of PI3KC2β by more than 80%, as compared to scrambled shRNA (Supplementary Figure S5B), in both WERI Rb1 and Y79 cells. In parallel we observed by CCK-8 assay that cell growth was reduced from 60 to 90% in WERI Rb1 (Supplementary Figure S5C) and from 75 to 95% in Y79 (Supplementary Figure S5D), upon suppression of PI3KC2β, as compared to scrambled shRNA, implying that PI3KC2β might play an important role in regulating retinoblastoma growth and survival.

## DISCUSSION

Despite the identification of loss or inactivation of the *RB1* gene as the main genetic driver responsible for the development of retinoblastoma, recent studies have suggested that additional pathways and genes are involved [31]. Here, we focused on activity of Notch in promoting growth and proliferation in retinoblastoma, since this pathway is known to play a key role in the specification and survival of stem and progenitor cells during retinal development [13]. In addition, in many types of malignancy Notch activation has been associated with an increase in overall growth and invasive capacity, as well as in maintaining a cancer stem cell population and inducing clonogenicity [32, 33]. Notch pathway has been recognized as a potential therapeutic target in several cancer types, such as pancreatic cancer, hepatocellular carcinoma, and glioblastoma [34–36].

Notch plays a crucial role in retinal development, since Notch1 is required to suppress photoreceptor differentiation and to maintain their progenitor state [10]. Deletion of Notch1 postnatally in mice promotes rod photoreceptor differentiation, whereas ablation of the receptor during early embryonic development enhances cone photoreceptor production [10]. *Jagged* was expressed in distinct regions within the optic vesicle, ciliary body, and lens, with patterns that changed over time during retina development in rat [20, 21]. Recently, it has been shown that Notch1 signaling may play a significant role in retinal ganglion cell development, with Notch1 positive progenitor cells able to differentiate into either ganglion cells or photoreceptors [37].

Interestingly, recent studies noted that Notch pathway components are differentially expressed in human tumors and in a murine model of retinoblastoma as compared to normal retina [22, 23]. In addition, Xiao et al. showed that Notch1 and Jag2 were highly expressed in the SO-Rb50 human retinoblastoma cell line compared to human retinal pigment epithelial cells, and that the cells were sensitive to the GSI DAPT [38]. A recent study showed that microRNA MiR-433 inhibits retinoblastoma growth by suppressing Notch1 and PAX6 expression [39]. We built on these observations and directly investigated the functional role of Notch in the regulation of overall growth and clonogenicity in two retinoblastoma cell lines, WERI Rb1 and Y79, using both a pharmacological and a genetic approach to suppress Notch pathway. In addition, we analyzed the effects of the Notch signaling inhibitor MRK003 on growth and apoptosis in combination with current chemotherapy, as a potential new therapeutic approach in retinoblastoma.

We found that the Notch target Hes1 was highly expressed in most primary tumor samples, and all key pathway components were also present in both retinoblastoma lines examined. The Notch ligand Jag2 was a major driver of pathway activity in these cells, as it was more highly expressed at the mRNA and protein levels as compared to other ligands and to non-neoplastic adult retina, and its suppression resulted in decreased receptor activation. However, adult retina is different from that of infants, and it therefore has some limitations as a control.

Pharmacological suppression of Notch signaling with MRK003, as well as genetic downregulation of Jag2 ligand or the canonical signaling cofactor CBF1, dramatically inhibited proliferation and overall growth and in both lines, as well as potently inhibiting colony formation *in vitro*, but did not reduce invasion. Downregulation of the ligand DLL4 also slowed growth of the tumor cells. These findings support an important role for Notch in promoting retinoblastoma pathogenicity.

Analysis of gene expression data indicated that the most common downregulated gene in both WERI Rb1 and Y79 lines, upon suppression of Jag2 by shRNA, was *PI3KC2β*, whose mRNA levels were decreased about two fold when the expression of Jag2 was suppressed by shRNAs. Since *PI3KC2β* is known to play a role in neuroblastoma tumorigenesis, by promoting Akt activity [30], its downregulation might be at least in part responsible for the potent inhibitory effect on growth and clonogenicity that we observed after suppressing Jag2 in both retinoblastoma lines. Indeed, suppression of *PI3KC2β* expression using shRNA slowed the growth of the tumor cells. Non-canonical Notch activity could be responsible for the growth inhibitory effects that we observed in the retinoblastoma cells after suppressing Jag2 ligand, particularly given the modest changes in some known canonical targets of the Hes and Hey families.

Upregulation of the cytokine receptor pathway was also observed when we combined the gene expression data from both retinoblastoma lines after Jag2 suppression, raising the possibility that this pathway might also play a role in promoting retinoblastoma growth downstream of Notch. Previous studies have shown that the CXCR4 receptor is expressed in the inner segment of photoreceptors, but its biological function in photoreceptor activity is not clearly understood [40].

Since chemotherapy represents a mainstay of treatment for retinoblastoma, we studied the effects of GSI in addition to Melphalan, and found that combinatorial treatment was more effective in inducing apoptosis and reducing cell survival in both retinoblastoma lines compared to single agents. Combination of Notch pathway inhibitors with standard chemotherapy might be a useful strategy in order to reduce the doses of the chemotherapeutic agent administered systemically or locally, limiting toxicity and side effects. High doses of Melphalan can induce several ocular side effects, including iris atrophy, retinal detachment, and vitreous hemorrhage, leading to possibly sight-threatening complications [41]. Other chemotherapeutic agents used in the treatment of retinoblastoma include Carboplatin, which is associated with nephrotoxicity and ototoxicity, and topoisomerase II inhibitors, such as Etoposide and Teniposide, associated in some cases with the appearance of secondary leukemias [42–44]. Therefore, new therapeutic regimens that improve vision retention and minimize adverse drug reactions represent potentially important advances in the treatment of retinoblastoma.

## MATERIALS AND METHODS

### Cell cultures, plasmids and chemical reagents

WERI-Rb1 [45] and Y79 [46] human retinoblastoma cells lines were obtained from American Type Culture Collection (ATCC, Manassas, VA) and cultured in RPMI-1640 supplemented with 50 IU/ml penicillin, 50 μg/ml streptomycin, 1% L-glutamine and respectively 10% or 20% heat-inactivated fetal bovine serum (FBS), at 37°C in a humidified 5% CO_2_ atmosphere. R28 cell line, a non-tumorigenic immortalized retinal cell line, derived from postnatal day 6 Sprague-Dawley rat retina [47], was obtained from Kerafast Inc. and cultured in DMEM 10% FBS medium. Retinal tissues resected from autopsy adult eyes at the Johns Hopkins Hospital were used as control to compare the expression of the Notch components in the retinoblastoma cells. Post-mortem time can affect RNA quality, and the autopsy eyes were received and processed within less than 18 hours of death. pLKO.1 transfer vectors containing short hairpin RNA (shRNA) targeting respectively Jag2 or CBF1 mRNA, whose target sequences were previously described [48, 49], were purchased from Thermo Fisher Scientific (Waltham, MA). Clones TRCN0000033415 and TRCN0000033418 were used to suppress DLL4 mRNA expression [50] and were purchased from Sigma-Aldrich (St. Louis, MO). pLKO.1 vectors with shRNA targeting PI3KC2b were obtained from GE Dharmacon (#RHS4533-EG5287, Lafayette, CO). Lentiviral particles carrying these constructs were prepared using HEK293T cells as previously described [48]. Puromycin (1 μg/mL) was used to select cells expressing the transfer vector. Scrambled or luciferase shRNAs were used as control in Jag2 loss-of-function experiments respectively in Y79 and WERI Rb1 cells. Scrambled shRNA was used as control in the CBF1, DLL4 and PI3KC2β loss-of-function experiments. The **γ**-secretase inhibitor (GSI) MRK003 was provided by Merck & Co., Inc. (Whitehouse Station, NJ) and dissolved in DMSO at the stock concentration of 10 mM [24]. Melphalan was purchased from Sigma-Aldrich (#M2011) and dissolved in DMSO at 10 mM as stock solution, following manufacturer's protocol.

### RNA extraction and quantitative real-time PCR

RNA extraction from retinoblastoma cell lines was performed using RNeasy mini kit (Qiagen, Germantown, MD). On-column DNA digestion was carried out with RNase-free DNase kit (Qiagen), to avoid contamination by genomic DNA. Quantitative real-time PCR (qPCR) for Notch pathway components was performed as previously described, using primer sequences previously published [48]. Primers for DLL1,3,4 and PI3KC2β were designed using Primer3 software: DLL1: 5′-TGCAACCCTGGCTGGAAA-3′ (forward), 5′-AATCC ATGCTGCTCATCACATC-3′ (reverse); DLL3: 5′-GAGA CACCCAGGTCCTTTGA-3′ (forward), 5-CAGTTGG AGCCTTGGAAACC-3′ (reverse); DLL4: 5′-GCG AACAGAGCCAGATTGAG-3′ (forward), 5′-CGCTCGT TGATGAACTCCTG-3′ (reverse); PI3KC2β: 5′-AGGCC CTGAGTCTTCTGTTC-3′ (forward), 5′-TGGTGATC TCAGGGGTTTCC-3′ (reverse). All reactions were carried out in triplicates and each experiment was repeated three times in iQ5 Multicolor real-time PCR detection system (Bio-Rad, Hercules, CA), using SYBR Green (Applied Biosystems, Foster City, CA) as fluorescent dye, and normalized towards β-actin mRNA levels.

### Protein analysis

The protein levels of Jag1, Jag2, cleaved/active form of Notch1 (NICD1), CBF1, and cleaved poly (ADP-ribose) polymerase (PARP) were determined by Western blot in the retinoblastoma cells. Proteins were extracted using TNE lysis buffer (50 mM Tris-HCl, pH 7.4; 150 mM NaCl; 5 mM EDTA; 1% SDS) supplemented with phosphatase inhibitor cocktail diluted 1:100 (Sigma-Aldrich), and 1 mM sodium orthovanadate (New England, BioLabs Inc., Ipswich, MA). The protein lysates were sonicated for 20 seconds and kept on ice. Protein concentration was measured using BCA kit (Pierce, Rockford, IL). Equal amounts of proteins were analyzed by 4–12% SDS–polyacrylamide gel electrophoresis (Invitrogen, Carlsbad, CA), transferred on a nitrocellulose membrane (Invitrogen) and incubated for 1 hour in a blocking solution containing 5% dried milk in TBS with 0.1% Tween 20 (TBS-T). The following primary antibodies were used during overnight incubation in blocking solution at 4°C: Jag1 (in rabbit, 1:800, Cell Signaling Technology, #2155, Danvers, MA), Jag2, (in rabbit, 1:800, Cell Signaling Technology, #2210), NICD1 (in rabbit, 1:800, Cell Signaling Technology, #4147), CBF1 (in rabbit, 1:800, Cell Signaling Technology, #5313), cleaved PARP at Asp^214^ (in rabbit, 1:800, Cell Signaling Technology, #5625), β-Actin (in mouse, 1:500, Santa Cruz Biotechnology, # sc-47778, Dallas, TX). Secondary antibodies bound to peroxidase and raised in mouse or in rabbit (KPL, Gaithersburg, MD) were used to visualize protein bands. Enhanced chemiluminescence (ECL) was used as detection reagent (PerkinElmer, Waltham, MA).

### Immunohistochemistry

Immunohistochemical staining was carried out on 5-μm-thick sections of formalin-fixed paraffin-embedded surgical specimens derived from enucleated eyes resected from patients diagnosed with retinoblastoma at the Johns Hopkins Wilmer Eye Institute, with local institutional review board approval. Standard techniques of immunostaining were utilized, as previously described [51]. In brief, slides were incubated overnight at 4°C with anti-Hes1 rabbit monoclonal antibody (Abcam, #ab108937, Cambridge, MA), diluted 1:500 in 2% normal goat serum/0.1% Triton X100/TBS. Secondary antibody was purchased from Vector Laboratories, Burlingame, CA (anti-rabbit: #PK-6101) and diluted 1:200 in 2% normal goat serum/0.1% Triton X100/TBS. Nuclear Hes1 immunoreactivity was scored in 11 samples by a board certified pathologist (C.G. Eberhart), and the intensity of staining scored as 0, no expression; 1+, weak expression; 2+, moderate expression; 3+, strong expression in over 50% of the cells. Human fetal brain was used as a positive control for Hes1 staining.

### Cell growth, viability, proliferation and soft agar assays

#### Cell growth assay

Cell growth was measured using Cell Counting-Kit 8 (CCK-8, Sigma-Aldrich), containing WST-8 reagent [2-(2-methoxy-4-nitrophenyl)-3-(4-nitrophenyl)-5-(2,4-disulfophenyl)-2H-tetrazolium, monosodium salt]. Cells were seeded at 10^4^ per well in a 96-well plate and resuspended in 200 μL of 5% FBS medium per each well. Cell viability was determined at the day of seeding (t_0_) and after 3, 6, 8 days of incubation at 37°C, by adding 20 μL of CCK-8 solution to each well and measuring the absorbance at 490 nm in a microplate reader (BioTek, Winooski, VT), after incubation for 2 hours at 37°C. For R28 retinal cells that grow in adherent conditions, cell growth was determined by MTS assay, as previously described [49], after 3, 6, 8 days of treatment with MRK003. Each experimental condition has been repeated in triplicate and data are presented as mean ± standard deviation (SD).

#### Cell viability

Viable cell number was measured by trypan blue exclusion dye after treatment of the cells for 5 days with MRK003 or Melphalan alone or in combination. CompuSyn software [52] was used to determine the Combination Index (CI) to define the interaction between the two drugs: CI<1, =1, >1 indicate respectively synergistic, additive, and antagonistic effects [53]. Exposure to γ-radiation was carried out using a Gammacell 3000 Elan (MDS Nordion, Ottawa, Ontario) and cell viability was determined 3, 5, 7 days post-irradiation by trypan blue exclusion dye.

#### Proliferation assay

The ability of the cells to replicate was determined by Ki67 incorporation assay, using Muse^®^ Cell Analyzer (Millipore, Billerica, MA), according to the manufacturer's instructions for non-adherent cells.

#### Soft agar clonogenic assay

Briefly, 1× 10^4^ retinoblastoma cells were mixed with RPMI-1640 medium, supplemented with 5% FBS and 0.5% agar (Invitrogen), and placed over a basal layer, containing 1% agar, in a six-well plate. MRK003 at 1, 3, 5 μM or DMSO were added to each layer. Cultures were incubated for 30 days at 37°C in a humidified 5% CO_2_ atmosphere, and then stained overnight to visualize the colonies with 1 mg/mL p-Nitro Blue Tetrazolium Chloride (NBT, Usb, Cleveland, OH) solution. Colonies greater than 100 μm in diameter were scanned and counted using MCID Elite software (Cambridge, England, UK).

### Apoptosis and invasion assays

#### Apoptosis assay

The apoptotic induction of retinoblastoma cells treated with Melphalan alone or in combination with MRK003 was determined by immunofluorescence, using cleaved caspase-3 primary antibody (in rabbit, 1:400, Cell Signaling Technology, #9661), as previously described [54].

#### Invasion assay

Cellular invasion was studied using transwell invasion assays as previously described [49], but slightly modified for non-adherent cells. Briefly, 6.5-mm-diameter Falcon cell culture inserts (8 μm pore size; Becton Dickinson, Franklin Lakes, NJ) were precoated for 1 hour with Matrigel (Becton Dickinson), diluted 1:100 in 10% FBS medium, in 24-well plates. 3 × 10^5^ cells were resuspended in 500 μL of serum-free RPMI-1640 medium and placed in the upper chamber, while 800 μL of medium containing 10% FBS were added in the lower chamber. After incubation for 48 hours, cells that had migrated through the filter floated in the medium located in the lower chamber. The amount of viable floating cells was measured in this chamber using CCK-8 assay: 80 μL of CCK-8 reagent were added to 800 μL of medium, after removing the insert, and the absorbance was read after 2 hours of incubation at 37°C. Data indicate the mean (± SD) of the absorbance at 490 nm in each of three independent experiments.

### Microarray analysis

Microarray assay was performed with SurePrint Agilent human GE 4× 44K version 2 one-color expression array (Agilent Technologies, Santa Clara, CA), using mRNA extracted from WERI Rb1 and Y79 lines transduced with two different Jag2 shRNAs (#1, #2), or with scrambled or luciferase shRNAs, along with parental cells. RNA quality was assessed by bioanalyzer (2100 Bioanalyzer, Agilent) and 400 ng of RNA was used for cDNA synthesis. Array hybridizations were carried out at the Microarray Core Facility of the Sidney Kimmel Cancer Center in Johns Hopkins University. Microarray raw data were analyzed using GeneSpring 12.6 software (Agilent) and *P* values were calculated using Moderated t-Test. *P* values lower than 0.05 were considered statistically significant. Ingenuity^®^ Pathway Analysis (IPA^®^) software (Qiagen) was used to determine the main signaling pathways modulated by the genes overexpressed or downregulated upon suppression of Jag2 ligand, compared to control shRNAs and parental lines. *P* values in Table 1 were calculated using Fisher's Exact Test.

### Statistical analysis

Experiments were carried out in biological triplicates and data are presented as the mean ± standard deviation (SD). Levels of significance were determined by 2-sided Student t test, with *p* values lower than 0.05 considered statistically significant. Statistical calculations were carried out using GraphPad Prism5 software.

## SUPPLEMENTARY MATERIALS


