# Supplementary material for: Targeting Notch signaling as a novel therapy for retinoblastoma
Source: Oncotarget. 2016 Sep 20;7(43):70028–44. doi: 10.18632/oncotarget.12142 (PMC5342532; doi:10.18632/oncotarget.12142)
Supplement: Supplementary file 1 [file oncotarget-07-70028-s001.pdf]

Targeting Notch signaling as a novel therapy for retinoblastoma

Supplementary Materials

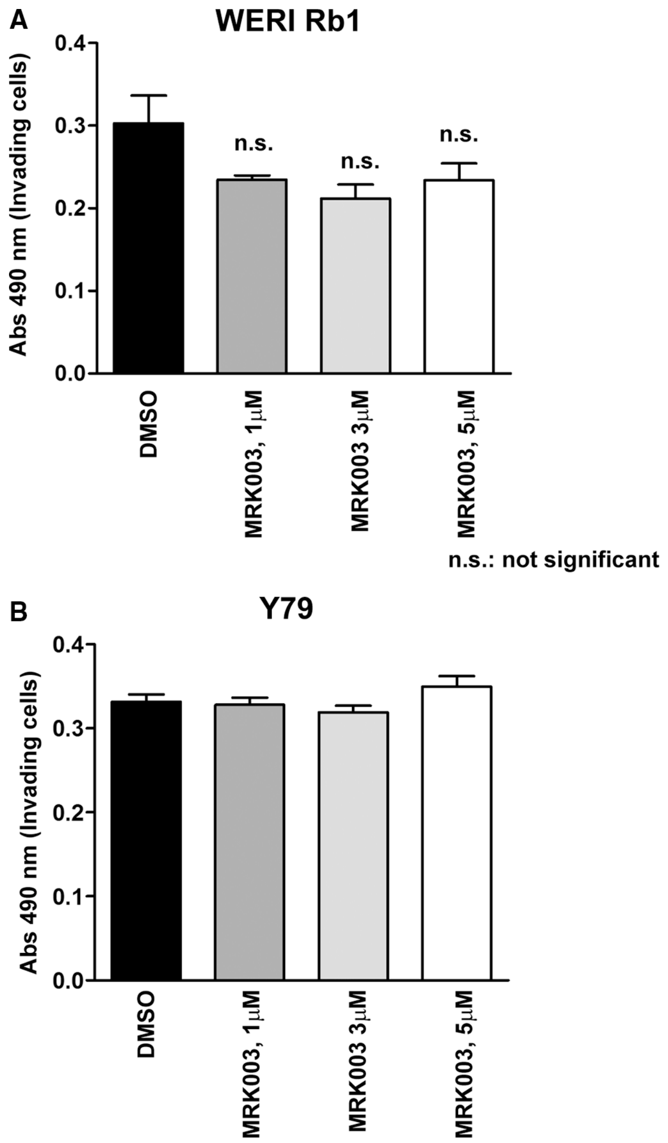

Supplementary Figure S1: MRK003 treatment did not modify invasion in retinoblastoma cells. (**A**), (**B**) WERI Rb1 (A) and Y79 (B) cells were treated with MRK003 at 1, 3, 5 µM for 48 hours. Invading cells were determined by transwell invasion assay using CCK-8 reagent. Data indicate the mean (± SD) of the absorbance at 490 nm, proportional to the number of invading cells, of three independent experiments.

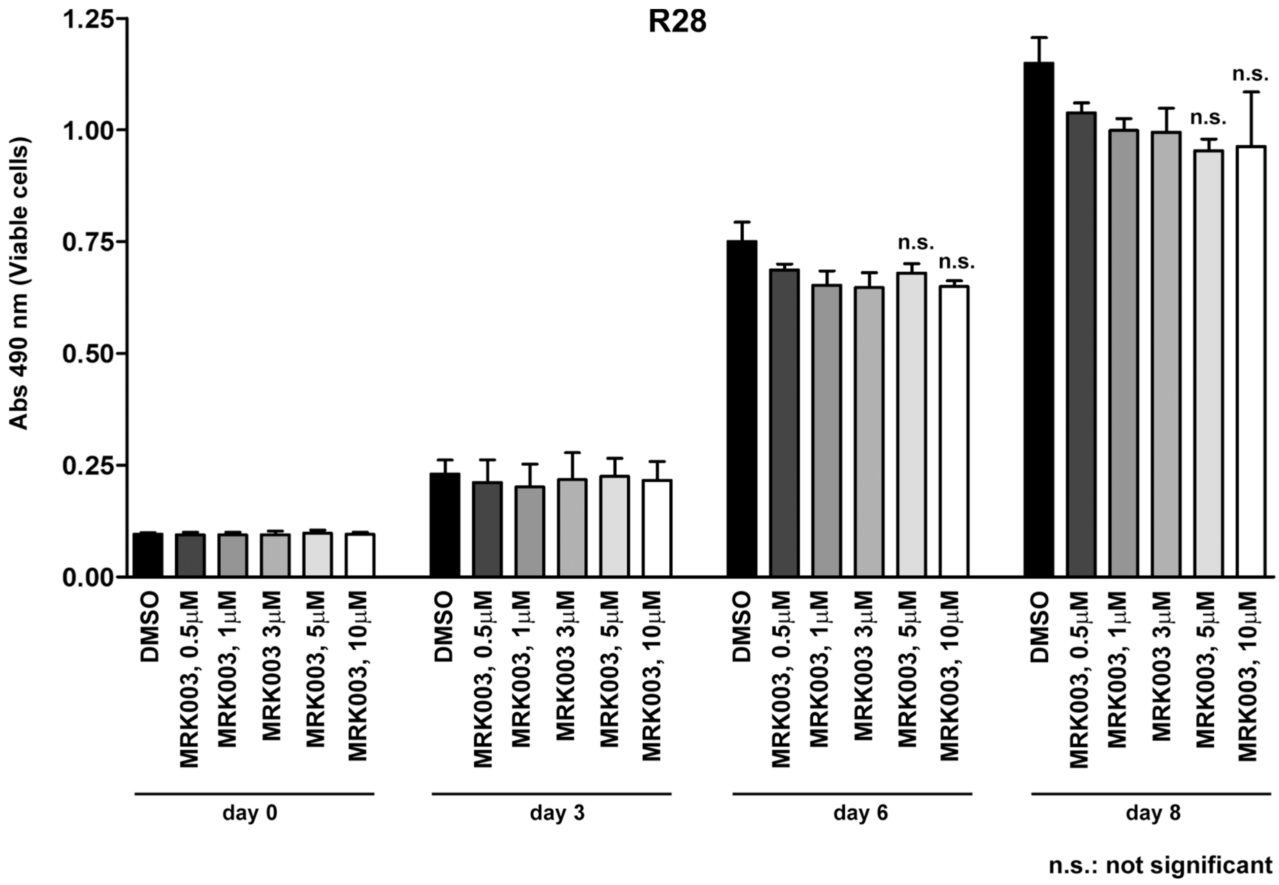

Supplementary Figure S2: MRK003 did not modify growth in R28 non-transformed retinal cell line. Cell growth was determined by MTS assay in R28 cells after 3, 6, and 8 days of treatment with MRK003 at 0.5, 1, 3, 5, 10 μM.

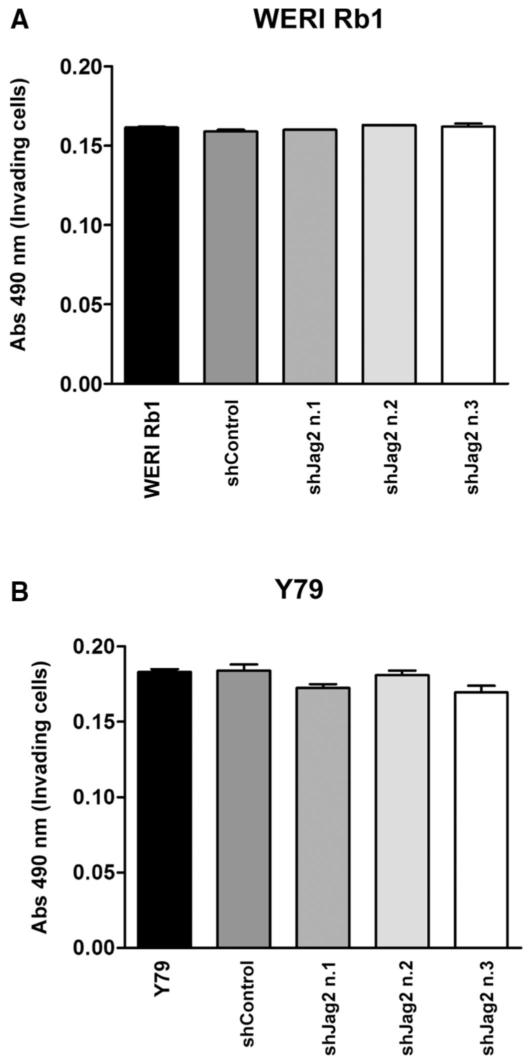

Supplementary Figure S3: Genetic downregulation of Jag2 did not modify invasion in retinoblastoma cells. (**A**), (**B**) WERI Rb1 (A) and Y79 (B) cells were infected with Jag2 or control shRNAs. Cell invasion was evaluated 48 hours after seeding the cells, by transwell invasion assay and the amount of invading cells was determined using CCK-8 reagent. Data indicate the mean (± SD) of the absorbance at 490 nm, indicative of the invading cell number, of three independent experiments.

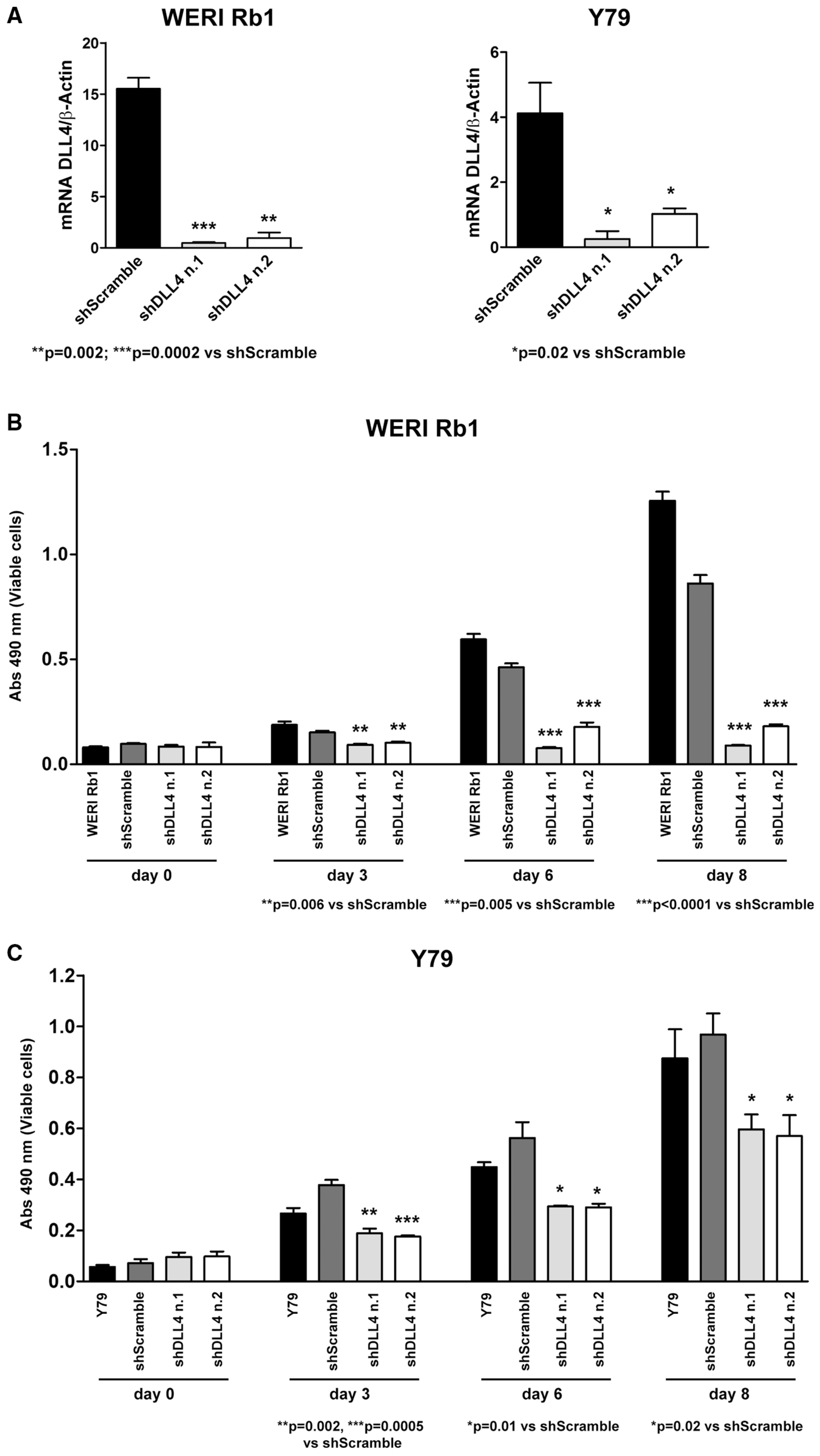

Supplementary Figure S4: Genetic downregulation of the Notch ligand DLL4 inhibits growth of retinoblastoma cells. (**A**) The mRNA expression of DLL4 ligand was suppressed by two separate shRNA constructs in WERI Rb1 and Y79 cells, compared to scrambled shRNA control, as found by qPCR. (**B**), (**C**) Cell growth was significantly reduced in WERI Rb1 (B) and Y79 (C) cells expressing DLL4 shRNAs compared to scrambled shRNA control, as found by CCK-8 growth assay.

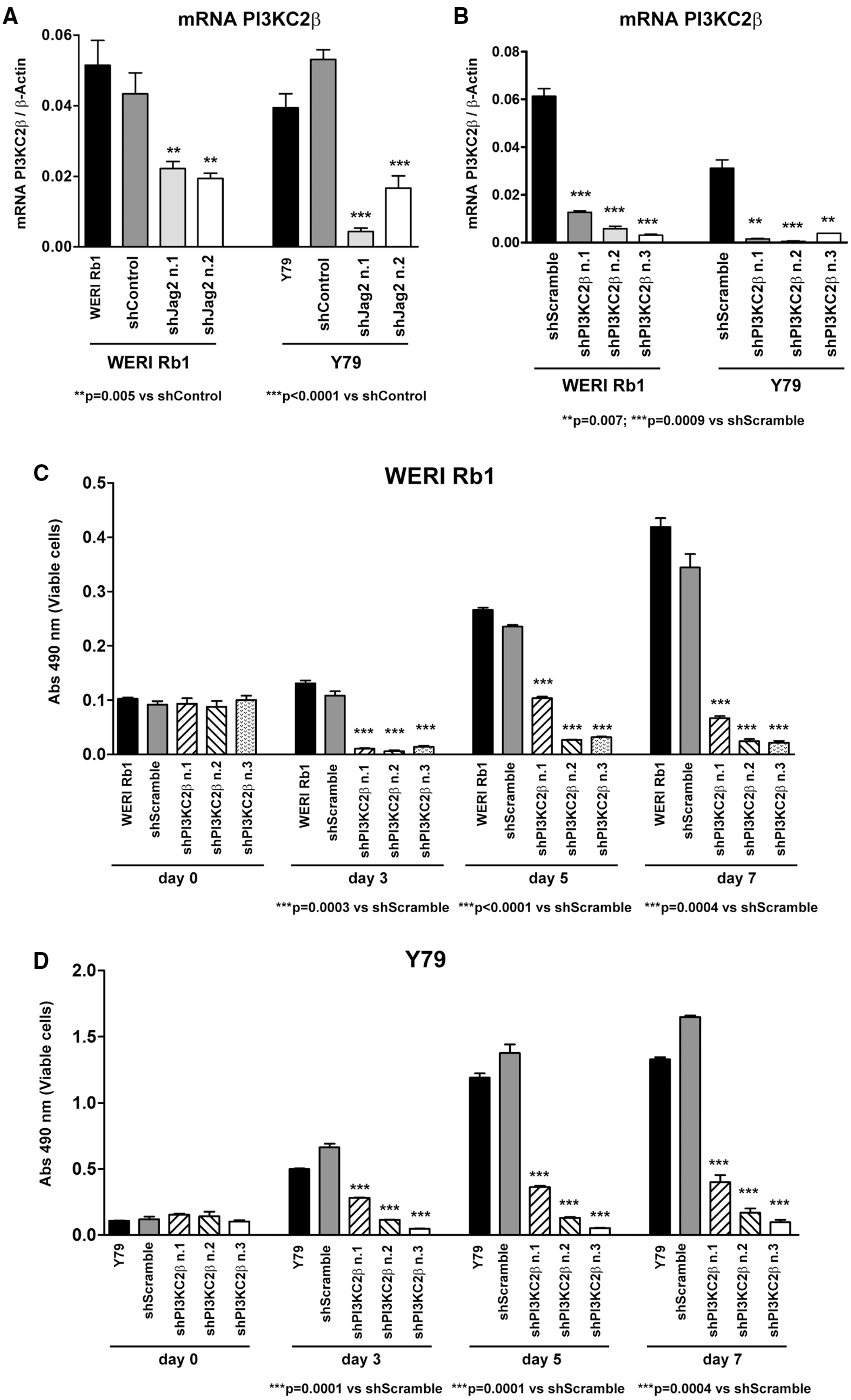

Supplementary Figure S5: Genetic downregulation of PI3KC2β suppressed growth of retinoblastoma cells. (**A**) The mRNA levels of PI3KC2β were determined by qPCR in WERI Rb1 and Y79 cells upon suppression of Jag2 by shRNA. (**B**) The mRNA expression of PI3KC2β was suppressed by three separate shRNA constructs in WERI Rb1 and Y79 cells, compared to scrambled shRNA control, as found by qPCR. (**C**, **D**) Cell growth was significantly reduced in WERI Rb1 (C) and Y79 (D) cells expressing PI3KC2β shRNAs compared to scrambled shRNA control, as found by CCK-8 growth assay.
